# Novel Avenues for the Detection of Cancer-Associated Viral Genome Integrations Using Long-Read Sequencing Technologies

**DOI:** 10.3390/cancers17111740

**Published:** 2025-05-22

**Authors:** Larissa-Anna Bergmann, Alicja Pacholewska, Michal R. Schweiger

**Affiliations:** 1 Institute for Translational Epigenetics, Faculty of Medicine, University Hospital Cologne, University of Cologne, 50931 Cologne, Germany; lbergma3@smail.uni-koeln.de (L.-A.B.); alicja.pacholewska@uni-koeln.de (A.P.); 2 Center for Molecular Medicine Cologne (CMMC), Faculty of Medicine, University Hospital Cologne, University of Cologne, 50931 Cologne, Germany; 3 Cologne Center for Genomics (CCG), West German Genome Center (WGGC), University of Cologne, 50931 Cologne, Germany

**Keywords:** HPV, human papillomavirus, LR-Seq, long-read sequencing, viral genome integration

## Abstract

Human papillomaviruses (HPVs), like many other viruses, are able to integrate their genomes into the host cellular genome. This integration can activate viral oncogenes or alter the function of cellular oncogenes and tumor suppressor genes, thereby increasing the likelihood of HPV-associated tumor development. In particular, HPV types 16 and 18 are responsible for over 70% of all cervical, anal, and oropharyngeal cancers worldwide, with rising incidence. Even more, high-resolution mapping of preferred integration sites using LR-Seq technologies offers deep insights into the molecular mechanisms of HPV integration. LR-Seq enables the detection of complex integration patterns, where the viral genome can be replicated and amplified into virus–host concatemers, including events within large structural variations or highly repetitive genomic regions. Furthermore, aligning LR-Seq data to the latest T2T reference genome (hs1) is necessary to provide new information about viral integration in genomic regions that were previously inaccessible, such as centromeres and other structurally complex repeat-rich loci. In this review, we provide insights into HPV genomic integration revealed by LR-Seq technologies, with a particular focus on how the use of the complete T2T reference genome enhances the detection of integration events in previously uncharacterized, repeat-rich regions of the human genome.

## Simple Summary

High-risk human papillomaviruses (HR-HPVs), especially types 16 and 18, contribute to the development of various cancers by integrating their genomes into the host DNA, activating oncogenes or disrupting tumor suppressor genes. This review discusses the limitations of short-read sequencing (SR-Seq) for integration analyses and emphasizes the importance of using long-read sequencing (LR-Seq) technologies, which enable high-resolution mapping of complex integration events, including those previously inaccessible for analysis, i.e., repeat-rich genomic regions. This review highlights recent findings from LR-Seq studies on HPV integration and compares its mechanisms with those used by other viruses.

## 1. Introduction

Human papillomaviruses (HPV)-associated carcinomas represent a large fraction (over 70%) of anogenital and oropharyngeal squamous cell carcinoma (OPSCC) in woman as well as in men [1]. Persistent infection with high-risk (HR)-HPV types can lead to viral genome integration, which can cause the development of various carcinomas, including head and neck squamous cell carcinomas (HNSCC), cervical carcinomas (CC), and anal, vulvar, penile, and vaginal carcinomas. Integration of viral genes can lead to altered cellular gene expression in or in close proximity to integration sites. Recent studies based on short-read sequencing (SR-Seq) revealed that the viral genome can directly integrate into coding regions, often within oncogenes or tumor suppressor genes [2]. SR-Seq technologies have enabled identification of common integration hotspots and recurrent integration sites, mechanisms of viral integration, and the functional consequences [3]. Although many discoveries have been made about the structure and mechanisms of the viral integration based on SR-Seq approaches, the availability and advancement of LR-Seq technologies have further improved the understanding of viral integration. Long-range structural variations, rearrangements, focal amplifications, and complex viral–host hybrid structures, induced upon integration, can efficiently be resolved using the advantages of long reads compart to short reads [4]. Another advantage of LR-Seq, in combination with the most recently elucidated complete telomere-two-telomere (T2T) reference genome (hs1), is the possibility to investigate repetitive regions in the genome and their possible involvement in the viral integration [5,6]. Within this review, we provide an overview about the application of recent LR-Seq technologies in HPV integration analysis compared to short-read sequencing. We aim to highlight the benefits of LR-Seq for identification of complex integration events and their possible functional consequences as well as the improved resolution of repetitive regions.

## 2. Classification and Occurrence of Human Papillomaviruses

**Figure 1:**
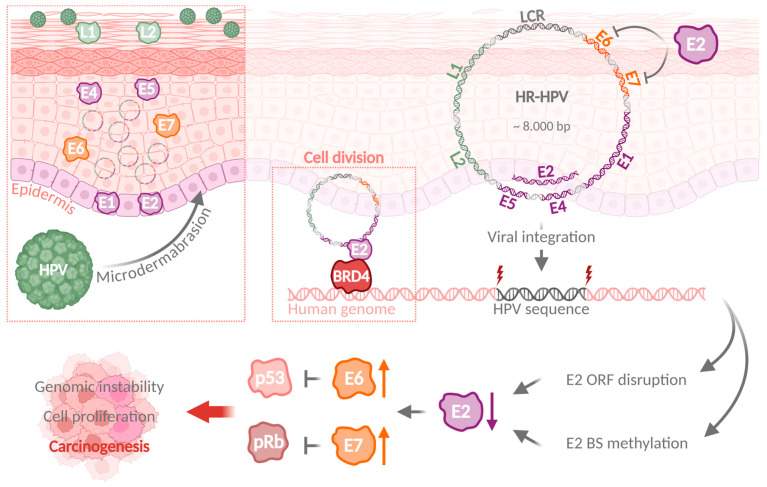
HPV infection can result in a viral genome integration and promotion of carcinogenesis. Upon HPV infection of basal epithelial cells through microdermabrasion, HPV proteins E1 and E2 are expressed in the infected cells, followed by increased expression of E6 and E7 synchronous to host cell differentiation. Viral genomes are replicated with high efficiency, until L1 and L2 facilitate virion assembly and release. HPV genome integration disrupts the E2 ORF, increases E6 and E7 expression, and results in p53 and pRb degradation and ultimately carcinogenesis. HPV: human papillomavirus; ORF: open reading frame; BS: binding site. (Created with BioRender.com).

HPVs are small, non-enveloped DNA viruses with a circular, double-stranded viral genome of approximately 8 kb. The viral genome encodes six early proteins (E1, E2, E4, E5, E6, and E7) responsible for virus replication and transcription as well as two late viral structural proteins L1 and L2. E1 is involved in origin recognition and replication and is supported by E2. Additionally, E2 proteins help in tethering the viral episomal genome to the host genome during mitosis and therefore are essential for viral replication and proper genome segregation [7,8]. E4 and E5 regulate the cell cycle and exert host immune evasion, respectively, to further promote viral replication and genome maintenance [9,10]. The HPV cell cycle regulator proteins E6 and E7 are considered as oncogenes due to their potential to degrade cellular tumor suppressors and to promote cancer development [11]. Upon infection, E6 degrades the tumor suppressor protein TP53 that is involved in DNA damage response (DDR) and cell cycle regulation. E7-mediated degradation of the retinoblastoma protein (RB1), which acts as cell cycle regulator and tumor suppressor, leads to genomic instability and promotes cell proliferation (Figure 1) [12]. Furthermore, genome instability is promoted by E6 and E7 through cell cycle deregulation, activation of DDR pathways, generation of oxidative stress, and telomere length alterations [13].

**Figure 2:**
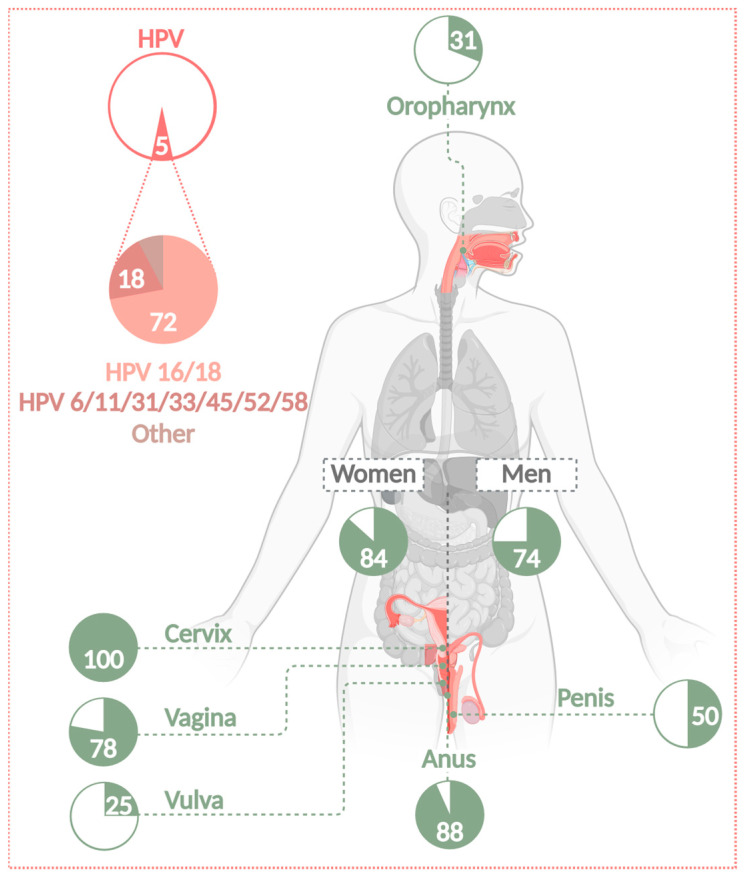
Number of cancer cases attributable to HPV infections. Almost 5% of all cancers worldwide are associated with an infection of HPV. HPVs can cause cervical, anogenital, and HNSSC (including OPSCC) in women as well as in men. The attributable fraction is 31% for OPSCC, 100% for CC, 78% for vaginal, 25% for vulvar, 88% for anal, and 50% for penile carcinomas. Of all these cases, 72% are caused by HR types 16 and 18. A further 18% are caused by infection with LR types 6 and 11 or HR types 31, 33, 45, 52, and 58 [1]. HR-HPVs cause 84% of all HPV-related cases in women and 74% in men [16] (Created with BioRender.com).

The specific HPV type and the site of infection can determine the clinical outcomes, ranging from benign warts to invasive cancers. Approximately 90% of all sexually active women and men are infected with one or more HPV types within their lifespan. Over 200 HPV genotypes have been identified and classified into five genera: alpha, beta, gamma, mu, and nu [14]. They exhibit different tissue tropisms and are associated with different clinical symptoms [9]. Of these types, alpha HPVs are the most common cause of disease, ranging from genital warts to various epithelial carcinomas. Alpha HPVs are further classified into low-risk (LR-HPV) and high-risk (HR-HPV) types, with the latter accounting for 5% of all human cancers worldwide [15]. At least 13 HR-HPVs are associated with cervical or anogenital cancers and head and neck carcinomas. Two of the most common high-risk types are HPV16 and 18, which account for 72% of all HPV-attributed cancers worldwide (Figure 2), followed by HR-HPV types 31, 33, 45, 52, and 58. The HR types account for 18% of the cases, together with the LR-HPV types 6 and 11 [1]. LR-HPV, like type 6 or 11, are considered to cause 90% of all benign warts, preferentially in genital regions.

## 3. Mechanisms of Viral Genome Integration Are Shared Between Different Oncogenic Viruses

HPV genomes are frequently integrated into the host genome. The viral integration of HR-HPVs results in an increased probability of developing HPV-associated tumors in the host, in most cases due to a loss of the E2 open reading frame (ORF) and an elevated expression of the viral E6 and E7 oncogenes (Figure 1). Genomic instability and accumulation of DNA double-strand breaks further promote viral genome integration [17]. Integration rates differ among various HPV types, ranging from around 50% of integrated viral HPV16 genomes to virtually complete integration of HPV18 genomes in CC samples [18]. Many studies have applied SR-Seq technologies to identify breakpoints and integration sites in HPV-associated tumors. They have provided a comprehensive tool to analyze and characterize viral integration and have identified integration sites in or in close proximity to oncogenes and tumor suppressor genes, i.e., genes associated with DNA damage repair or genomic instability [19,20]. Besides the deregulation of single cancer-related genes upon HPV infection, frequent integration into or in close proximity to genes associated with DNA damage response suggests the involvement of this pathway in the induction of genomic instability and HPV integration [21]. Additionally, this pathway provides a platform for integration through microhomology-mediated DNA repair, which is suggested to be one of the mechanisms for the HPV genome integration [22,23]. More recent studies using LR-Seq methods were able to elucidate viral integration events with even higher resolution compared to short-read sequencing. Complex viral–host interactions and long-range structural variations, including rearrangements, deletions, and translocations, were uncovered [4]. Additionally, these approaches precisely mapped integration events to more complex genomic regions, like repetitive sequences. Since different regulatory elements like transposable elements and enhancer- or repeat-derived super-enhancers can reside within these regions [24], deregulation may provide a growth advantage and promote viral persistence.

**Figure 3:**
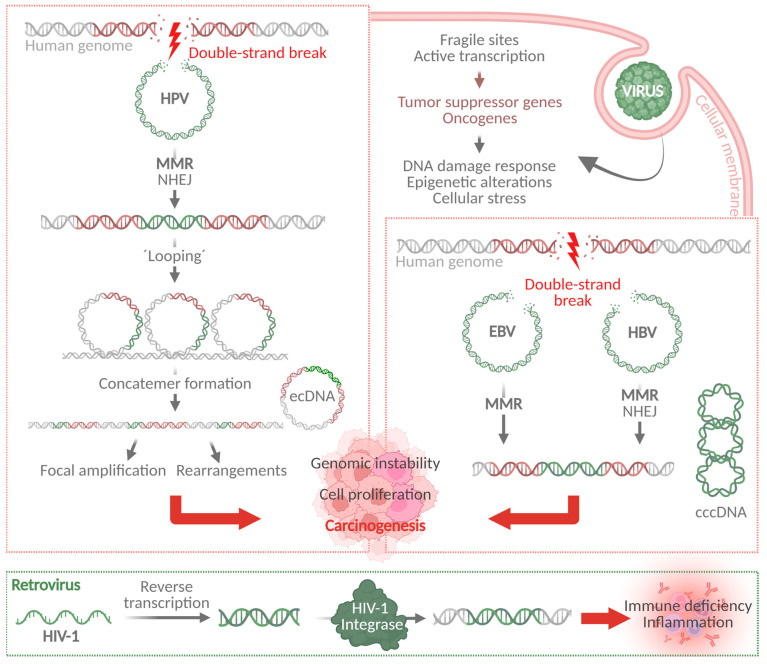
Viral integration mechanisms. Viral genomes are associated with fragile sites, harboring oncogenes and tumor suppressor genes and thus activating DNA damage response, epigenetic alterations, and cellular stress. DNA double-strand breaks occur, and viral genomes are integrated through MMR or NHEJ. These mechanisms are similar for HPV, EBV, and HBV. Besides integrating their genomes, HBV genomes form cccDNA after integration and HPV form ecDNA. HPV integration induces looping and concatemer formation, leading to focal amplifications and rearrangements, ultimately resulting in genomic instability, aberrant cell proliferation, and carcinogenesis. In contrast to this, retroviruses like HIV-1 reverse transcribe their viral RNA and facilitate integration using the HIV-1 integrase. This leads to immune deficiency and inflammation. HPV: human papillomavirus; MMR: microhomology-mediated repair; NHEJ: non-homologous end-joining; ecDNA: extrachromosomal circular DNA; cccDNA: closed covalent circular DNA; EBV: Epstein–Barr virus; HBV: hepatitis B virus; HIV-1: human immunodeficiency virus 1. (Created with Biorender.com).

Based on the observation of complex virus–host concatemers and rearrangements, including focal amplifications, excision, and reinsertion of viral–host sequences, integration was proposed to take place according to the “looping” model [23,25] (Figure 3). Furthermore, integration can be reversed, leading to the excision of virus–host fusion sequences, which can amplify, rearrange, and integrate again, existing as oncogenic extrachromosomal circular DNA (ecDNA) [4]. Many tumors exhibit a mixed pattern of integrated and ecDNA viral–host sequences [4,26,27]. HPV integration sites, as cancer-related genes, have been associated with structural variations in the human genome explaining the proximity of the HPV integration sites with the host cancer-related genes. These structural variation events are formed due to the rolling circle amplification that takes place at the integration breakpoint, leading to the formation of amplified segments of genomic sequences flanked by HPV segments [23,25,28,29]. A possible mechanism for the HPV genome integration could be the preferred integration within fragile sites and repetitive regions or near centromeres at early stages of carcinogenesis or lesions through pathways and machineries closely related to genomic instability [19]. Later, during carcinogenesis, a clonal selection could be the reason for an enrichment of cancer-associated genes further promoting cancer development.

Other oncogenic viruses, like Epstein–Barr virus (EBV), hepatitis B virus (HBV), hepatitis C virus (HCV), human T-cell lymphotropic virus 1 (HTLV-1), Kaposi sarcoma-associated herpesvirus (KSHV), or Merkel cell polyomavirus (MCPyV), exert similar functions on host gene expression after infection, including epigenetic changes and dysregulation of host genes, oncogenes, or miRNAs [30]. EBV, HBV, and HCV infections, similarly to HPV infection, lead to hypermethylation of promoters of important genes like *CDH1*, which encodes E-cadherin that promotes epithelial–mesenchymal transition (EMT) and subsequent carcinogenesis if repressed [31,32,33,34]. An increase in H3K27me^3^, histone modification repressive mark, is commonly observed after viral infection due to upregulation of the DNA (cytosine-5)-methyltransferase 1 (DNMT1). These mechanisms all contribute to EMT, metabolic reprogramming, apoptosis inhibition, genomic instability, and increased cell proliferation, ultimately resulting in carcinogenesis [30]. A recent study found similarities between the integration mechanisms of HPV, HBV, and EBV. This study found a shared proportion of an alternative end-joining mechanism upon double-strand break-induced integration of viral genomes, which is probably mediated through microhomologies [35] (Figure 3). Interestingly, an infection with the latent human immunodeficiency virus 1 (HIV-1) was found to be associated with integration into transcriptionally inactive and repetitive centromeric satellite regions to possibly evade the host immune response [36]. Oncogenic viruses, including RNA retroviruses like HIV-1 or HTLV-1, are supposed to drive clonal expansion upon genome integration, which is suggested to be non-random. Unlike in retroviruses, HPV viral genome integration is not a part of the viral life cycle [37] and rather possesses a dead-end for the virus [38]. However, clonal expansion is frequently observed in HPV-associated integration events, indicating a non-random integration mechanism [4]. Shared mechanisms between different integrating viruses open up new possibilities for the investigation of carcinogenic mechanisms for the development of new therapeutic targets that could be commonly applied.

## 4. Recurrent HPV Integration Sites Are Associated with Tumor Progression

Viral genome integration can cause precancerous lesions, which subsequently can develop into various cancers, including HNSCC, CC, anal cancer, and the less common vulvar, penile, and vaginal carcinomas. Integration of viral genes into the host genome can lead to a change in expression of cellular genes in or in close proximity to integration sites. In many squamous cell carcinomas, it was observed that the viral genome can directly integrate into coding regions, often containing oncogenes or tumor suppressor genes, which are then disrupted and that can result in carcinogenesis [2]. HPV integration in HNSSC was observed to occur with a relatively low frequency of approximately 13% compared to up to 80% in cervical squamous cell carcinomas [23]. Although integration rates differ between tumor entities, several studies detected common integration hotspots and recurrent integration sites [3]. It has been suggested that the site of integration may provide a cell-selective growth advantage rather than reflecting a specific target for an integration event [29,39]. However, several hotspots identified, like *MYC*, *RAD51B*, and *TP63* that are detected in CC as well as HNSCC [2,20,23], indicate a targeted rather than random integration mechanism. Fan et al. [40] observed a difference between the eventuality of silent and actively transcribed (productive) integration sites. The latter were found to be preferentially integrated into introns, common fragile sites, and repeat elements and lead to upregulated expression of host genes [40]. The observation that E6 and E7 oncogenes usually remain intact during integration further supports the hypothesis of a targeted integration mechanism [23]. It was long assumed that a breakpoint in E2 is necessary for de-repression of E6 and E7 to promote carcinogenesis, but recent studies based on more precise breakpoint mapping detected a broad and unspecific breakpoint pattern throughout the whole viral genome. Exceptions are the long control region (LCR) and E6 and E7 ORFs [23,25]. Furthermore, Chaiwongkot et al. [41] found a positive correlation between the number of HPV integrations and the methylation status of E2 binding sites. Through methylation of E2 binding sites, E2 function may be inhibited, which ultimately results in dysregulation and overexpression of E6 and E7, even without previous integration [41]. Additionally, HPV E2 proteins interacting with the cellular bromodomain-containing protein 4 (BRD4) were found to be associated with fragile sites during replication [42]. A close association of the viral genomes with fragile sites during replication could lead to an increased integration rate. Furthermore, during cell division the viral episomal genome is tethered to the host genome by BRD4 [8]. This mechanism could also serve as the basis for integration at specific sites since integration was previously associated with transcriptionally active regions [43,44] and fragile sites [45,46] in particular.

## 5. Technologies Can Identify Viral Integration Sites with Variable Resolution

For the investigation of HPV integration sites, various technologies are currently applied, ranging from PCR-based methods, SR-Seq, and more recently LR-Seq. Thereby, integration sites can be identified, and the mechanisms of HPV integration in HPV-associated cancers can be resolved [4,47]. Previously, frequent integrations in or in close proximity to *RAD51B* [2,19,20,48], *TP63* [2,20], *KLF5* [2,27], *MACROD2* [19,27], or *MYC* [2,4,49] were observed. These genes were also identified as hotspot genes with LR-Seq, validating the specificity and accuracy of the method [49]. However, non-sequencing-based technologies are still frequently applied to detect viral integration without the determination of the exact integration site. These methods include in situ hybridization to visualize the number of integration sites within the nucleus or multiplex human papillomavirus genotyping [50]. PCR-based approaches such as the ligation-mediated detection of integrated papillomavirus sequences (DIPS)-PCR [51] or amplification of papillomavirus oncogene transcripts (APOT)-PCR technologies can detect virus–human DNA sequences or virus–human RNA transcripts, respectively [52]. Both approaches are sensitive and cost-effective and can identify integration sites accurately. Whole-exome sequencing (WES) and whole-genome sequencing (WGS) can efficiently detect integration sites and their structure. However, they are mainly restricted to coding (WES) or non-complex regions (WGS) because mapping of short reads to complex, repetitive-rich regions is ambiguous. In addition, using WES and WGS for this application is relative expensive due to a need for sufficient coverage depth. Capture-based methods are frequently used prior to the methods mentioned above to reduce the costs. Higher sensitivity is reached due to HPV-targeted enrichment, but the identification of novel integration sites is limited [47]. RNA sequencing (RNA-Seq) can determine specific effects of the viral integration on host and viral gene expression as well as viral–host fusion transcripts in transcriptionally active regions, but it will disregard any integrations within intergenic regions [43]. Some RNA-based methods, like mRNA-Seq, are not suitable for the identification of lncRNA, miRNAs, or other small RNA in the context of HPV integration due to the lack of RNAs without polyA tails used for the library preparation. This limitation could be avoided by using random priming, with the caveat of introducing a potential coverage bias [29]. In contrast, LR-Seq approaches can resolve even more complex genomic structural variations and rearrangements caused by HPV integration. Usually, a lower sequencing depth and accuracy is required [4,26]. One caveat of these technologies until recently was the relatively high error rate. However, according to recent studies, the sequencing quality of Pacific Biosciences of California, Inc. USA (PacBio) [53] and Nanopore Technologies plc, Oxford, United Kingdom [54] has reached the quality of SR-Seq reads. Moreover, calling methylation changes using both LR-Seq technologies has also reached high accuracy, especially for well-covered bases [55]. Another challenge is the scarce availability of bioinformatics tools for analyzing LR-Seq data. Tools for SR-Seq based integration studies have been well established [56]. Like for SR-Seq, LR-Seq integration site analysis starts with detection of chimeric reads spanning the viral and host genome, either by filtering fused reads mapped to a synthetic reference genome containing the viral genome as an additional chromosome or by mapping the reads to both genomes separately. An example of a tool that can be applied for both SR-Seq and LR-Seq data for the detection of integration sites is VIRUSBreakend [57]. This tool allows for the annotation of the integration sites within repetitive regions and was used to show additional integration sites detected upon re-analysis with the complete T2T hs1 as the reference compared to previous analysis with GRCh37 [58].

## 6. LR-Seq Efficiently Resolves Structural and Functional Complexities of HPV Integration

**Figure 4:**
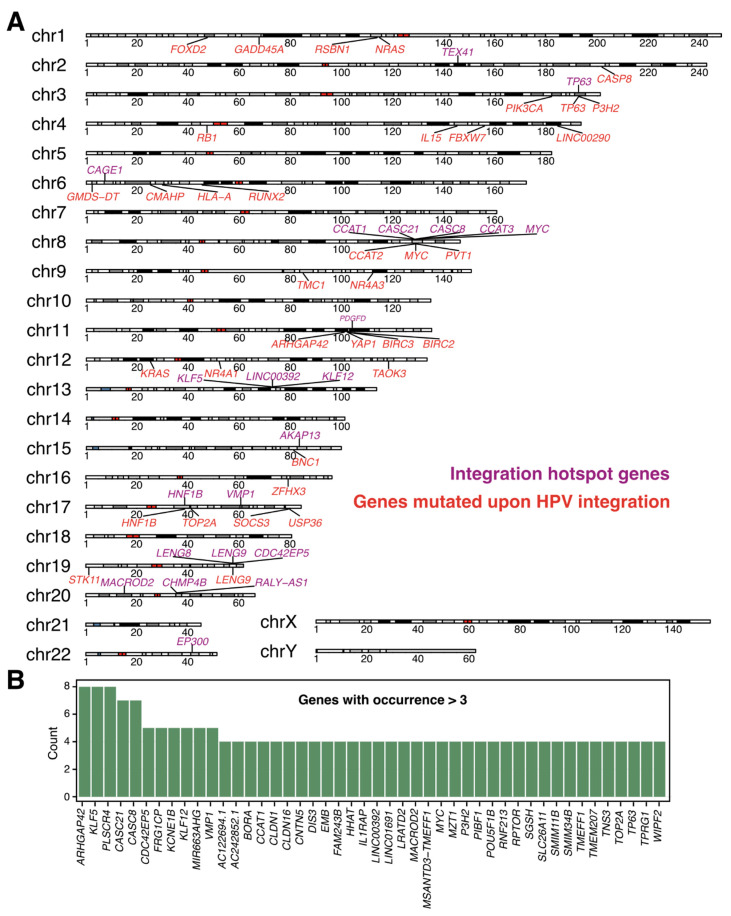
Comparison of integration events identified with LR-Seq. (**A**). Chromosomes with hotspot genes identified by LR-Seq are shown. Genes that harbor an integration site within or in close proximity to their locus are shown in violet, and genes that are mutated upon HPV integration are shown in red. (**B**). Bar plot showing the frequency of the hotspot genes compared between studies using LR-Seq. Integration data were compared, and the plotting cutoff was set to genes occurring more than two times in the LR-Seq studies from Table 1.

Within this review, we compare key findings from published studies investigating HPV integration specifically using LR-Seq. We provide a comprehensive overview about main insights, novel integration sites, and mechanisms in the context of HPV-associated cancer progression after viral integration. Table 1 summarizes recent publications on LR-Seq, and includes information on samples, sequencing methods, and the reference genome used. It also includes affected genes and genomic regions identified as integration sites. Different HPV-associated cancer samples were collected and analyzed, including tissue from HPV-positive HNSCC, oropharyngeal squamous cell carcinomas (OPSCC), or HPV-positive CCs. Furthermore, samples included HPV-positive cell lines, e.g., 93-VU-147T (HPV16+), CaSki (HPV16+), GUMC-395 (HPV16+), HeLa (HPV18+), HTEC (HPV16+), or SiHa (HPV16+) cells. The sequencing technologies used varied between the studies: nine studies used Oxford Nanopore, three studies used PacBio technology, and one study used PacBio and Illumina, San Diego, California, USA WGS. One study specifically used PacBio Iso-Seq for RNA sequencing. The HPV reference genome used varied between the studies and depended on the investigated HPV types. All of the studies either aligned their data to GRCh37 (*n* = 5; 42%) (GenBank GCA_000001405.1) [59] or GRCh38 (*n* = 7; 58%) (GenBank GCA_000001405.15) [60]. The hotspot genes listed in Table 1 refer to the integration sites identified by the individual studies as most prominent or that exhibited an enrichment of integration events. Some studies defined hotspot genes as genes that harbor an integration (I), while others define hotspots as genes carrying a mutation (M) after integration. All integration sites identified are summarized in greater detail in Figure 4. The genomic region column summarizes the regions where all integrations identified in the studies occurred. Overall, integration sites were enriched in introns, exons, and intergenic regions as well as in promotors, non-coding RNA (ncRNA), untranslated regions (UTRs), and CpG islands, to a smaller extent (see Table 1).

In particular, Wang et al. [49] recently described the complex integration structure in cervical cancer cell lines, assigned specific integration-associated structures to certain chromosomes, and revealed an altered gene expression profile after integration using Oxford Nanopore LR-Seq in HPV-positive cell lines. Compared to targeted-capture SR-Seq, additional information was provided about the structure of the integrated sequence and their influence on genomic structural variations, the rearrangements around the integration site, and their influence on gene deregulation. The observed structure of HPV integration was proposed to result from a specific form of DNA breakage, called breakage-fusion-bridge cycles, as a result of long-range chromosomal rearrangements induced through HPV integration [64]. In addition to the characterization of complex and long-range structural variations caused by HPV integration an evolutionary model for HPV integration and its clonal origin was developed [4,27]. In detail, Zhou et al. [27] demonstrated that LR-Seq is sufficient to resolve inter-chromosomal translocations. Akagi et al. [4] showed that integration can trigger replication instability, promoting clonal evolution and intratumoral heterogeneity. The use of Oxford Nanopore sequencing (and also PacBio HiFi chemistry) offers the possibility to additionally detect epigenetic changes upon HPV integration [26,61]. Human genomic regions upstream of HPV transcription were found to be hypermethylated, and downstream regions were hypomethylated [26]. Hypomethylation of the HVP LCR after integration was suggested to be a driver of E6 and E7 overexpression and subsequent carcinogenesis [61].

Some studies included the analysis of integration sites in HPV-positive cell lines like HPV18-positive HeLa cells and HPV16-positive SiHa or CaSki cells. They applied LR-Seq to identify diverse integration patterns and structural complexities, which varied between HPV types [49,62]. Thus, not only do cancer tissue samples have a relevance in resolving HPV integration patterns, but cell lines can also provide a comprehensive understanding of the underlying mechanisms and integration patterns.

Some of the studies selected used multi-omics approaches and combined SR-Seq and LR-Seq to identify integration sites with high accuracy and to provide a comprehensive characterization of long-range virus–human integration events. Both sequencing approaches and the identified integration sites were compared, and it was demonstrated that LR-Seq is able to resolve the complex structure of HPV integration events [4,49]. Akagi et al. [4] utilized LR-Seq and observed a complex structure of repetitive and interrelated concatemerized viral–host DNA hybrids, called “heterocateny”, upon integration in HPV-associated tumors. These structures suggest the presence of clonal evolution and promotion of heterogeneity in HPV-associated tumors [4]. Recombination between repetitive and homologous regions was proposed as one of the mechanisms for ecDNA formation and HPV integration [27]. Porter et al. [26] compared the detection of integration events between SR-Seq and LR-Seq and observed a higher confidence mapping for long reads compared to short reads within repetitive regions. Interestingly, they reported a good correlation (Spearman’s correlation, R = 0.78, *p* = 3.8 × 10^−15^) between the number of called integration events per sample using long-read and short-read sequencing, with similar proportions of calls mapped to exons, introns, and CpG islands [26]. Although SR-Seq allows for initial identification of integration sites and their validation, LR-Seq adds an improved resolution of the spatial and structural features of integration events [67]. The use of PacBio Iso-Seq further suggested that novel isoforms may result in the overexpression of E6 and E7 oncogenes in CC samples, driving cervical carcinogenesis [65]. The promotion of carcinogenesis using PacBio Iso-Seq for transcriptome profiling of a different cancer type, chronic lymphocytic leukemia (CLL), was efficiently demonstrated recently [69]. This further highlights the benefits of LR-Seq not only for HPV integration analyses but also for analyzing carcinogenesis in general and enables a functional analysis of oncogenic events.

Not only can structural changes be resolved accurately using LR-Seq, but the identification of deregulated, mutated, or affected genes upon HPV infection is also much easier. Long reads spanning several megabases can connect the integration sites to a specific mutation generated at a different locus and thus could provide an explanation for the development of structural variations and mutations upon HPV infection. A summary of the hotspot genes identified using LR-Seq, according to the studies mentioned in Table 1, in relation to their genomic locus is presented in Figure 4A. The presented genes are divided into genes that harbor an integration site within or in close proximity to their locus and genes that were found to be mutated or deregulated upon HPV integration. The hotspot genes were selected according to what the individual studies assigned as hotspots. Not every chromosome harbored an integration site or mutated gene, which could be due to the limited number of studies applying LR-Seq for HPV integration analysis. Besides, some chromosomes show an accumulation of integration events at specific loci. Further studies are needed to investigate these clusters at specific loci in greater detail. The clustering indicates the presence of hotspots for integration, which were previously observed [3]. Some studies mentioned an overlap between the identified hotspots using LR-Seq and short-reads and claimed that they used both methods to validate their results [4,49]. Zhao et al. [61] compared their LR-Seq data to previously published short-read data and observed an overlap for integrations sites in general as well as for specific hotspot genes. Additionally, they found new integration sites, which are probably the result of a better resolution of LR-Seq [49]. Comparison of all integration events and affected genes, identified by LR-Seq [26,27,61,63,64,66,67], also supports the presence of hotspot genes (Figure 4B). Top candidates like *KLF5*, *KLF12*, *MACROD2*, *MYC*, or *TP63* were already proposed as hotspots using SR-Seq [3]. This observation allows for the conclusion that HPV integration may not be random, and clonal evolution with HPV-associated tumors may possess a growth advantage, depending on the integration site. It further highlights the necessity to use LR-Seq for HPV integration analyses to efficiently resolve complex structures and mechanisms.

The resolution of integration sites improves when LR-Seq methods are used. Novel integration sites were detected while also previous ones were confirmed showing the liability of the methods [4]. LR-Seq can capture viral integration within complex structures, including large chromosomal rearrangements, tandem repeats, and inter-chromosomal translocations as well as epigenetic changes [26,49,62]. Full-length viral–host fusion transcripts can span several million bases, including repetitive regions, and they can provide a basis for the characterization of integration events and a functional analysis of carcinogenesis due to genome-wide transcriptional dysregulation [4,27,65]. In contrast, complex long-range structural variations are missed by SR-Seq, and it is not possible to resolve integration sites within repetitive regions with SR-Seq data due to ambiguous mappings of the reads. Nevertheless, SR-Seq remains valuable for high-throughput applications and quantification approaches such as transcriptomics analyses. Studies consistently emphasize the clinical potential of LR-Seq technologies for understanding HPV-driven carcinogenesis and tailoring therapeutic strategies [63,66,70]. Recent developments in LR-Seq have made the technology suitable for rapid data processing and a cost-effective clinical application due to a simplified workflow without the necessity for prior enrichment or error correction [66]. However, SR-Seq is useful for broader population studies or initial screenings where detailed integration structures are not the primary focus.

The presence of recurrent integration hotspots across several studies leads to the assumption that viral integration is non-random. LR-Seq can identify a certain proportion of novel integration sites, which leads to the assumption that, depending on the applied method, the data analysis and interpretation of the results are subjected to substantial variations. 

In summary, while direct studies on HPV integration using the T2T reference genome are currently lacking, the improved genomic coverage and accuracy of the T2T assembly suggests that future research in this area could benefit from adopting this reference to achieve more precise mapping of integration events, especially in previously unresolved and complex repetitive genomic regions. High-resolution identification of integration sites throughout the whole genome using LR-Seq approaches can provide a detailed understanding about the molecular mechanisms of viral integration events and preferred integration sites to aim for novel therapeutic targets, preventing HPV integration and subsequent tumor development. Within this review, we discuss integration events based on LR-Seq. However, the current data availability of LR-Seq, especially with regard to repetitive regions, is sparse, and therefore, conclusions about the mechanisms remain elusive. With the publication of the T2T reference genome, we expect increasing research in this field, which will allow for more precise conclusions about HPV or even viral integration in general. Moreover, this review focused mainly on HPV16- and HPV18-induced cancer types. However, using this information and integrating data about other viral integration mechanisms serves as a basis for the general understanding of integration mechanisms and future research targets.

## 7. HPV Frequently Integrates into Repetitive and Non-Coding Regions of the Host Genome

The human genome contains approximately 50% repetitive DNA sequences [71]. Repetitive sequences can be divided in tandem repeats, like human satellites or retrotransposons, long interspersed nuclear elements (LINEs), short interspersed nuclear elements (SINEs), long terminal repeats (LTRs), and other transposable elements [5,24,72]. Some repetitive regions, such as centromeric and pericentromeric satellite sequences, encode long non-coding RNAs (lncRNAs), and their increased expression is frequently found to be enriched in a broad range of various cancer entities, including epithelial carcinomas [73]. It is thought that lncRNAs contribute to oncogenic transformation by promoting chromosomal instability, DNA damage, and epigenetic alterations [74,75]. Upon HPV infection, lncRNAs could also promote genomic instability, subsequent viral genome integration, and carcinogenesis. Sharma et al. [76] found that around 20% of annotated lncRNAs were differentially expressed upon HPV16 E6 and E7 overexpression, which was due to viral genome integration. For example, changed expression of the lncRNAs *PVT1* or *MALAT1* in CCs was attributed to deregulation by E6 and E7 [76], which could induce genomic instability and promote viral genome integration. Another prominent fraction of non-coding RNAs are microRNAs (miRNA), which were also found to be differentially expressed and dysregulated in several HPV-associated cancers [77,78]. For example, miR-145 [79] and miR-34a [80] were shown to be involved in E6- and E7-related pathways and subsequent tumor progression. Interfering with these pathways could further promote genomic instability and viral genome integration. Besides the observed integration into known genes, especially oncogenes, several studies reported repetitive and intergenic non-coding regions as a possible target site for viral genome integration [3]. The assembly of short reads to repetitive regions is challenging, which can lead to errors and ambiguities in mapping viral integration sites. However, enrichment of integration sites in or near centromeric regions was previously observed, using a targeted high-throughput viral integration detection (HIVID) assay combined with SR-Seq [19]. Additionally, the centromere protein-C (CENP-C) was shown to interact with HPV E7, further suggesting a direct integration mechanism at repetitive regions, which might be facilitated through the interaction between HPV proteins and cellular proteins [81]. One well-studied interaction is the viral genome tethering to the host genome through a direct interaction between HPV E2 and BRD4, which brings both genomes in close proximity and could lead to viral integration at these sites [8]. Li et al. [82] used DIPS-PCR and found frequent integrations into or near repetitive genomic regions, such as LINE, SINE, and ALU sites in HPV16-positive CC samples. Furthermore, Porter et al. [26] detected 51% of integration breakpoints within repetitive regions using LR-Seq. In comparison to the short-read-based sequencing approaches, the use of LR-Seq enables the resolution of long viral integration and the assembly of repetitive regions [66].

In addition to the difficulties, which come along with mapping short reads to repetitive regions, previous studies used reference genomes, such as GRCh37 and GRCh38, which lack major parts of repetitive regions [6]. Only since the most recent full-length T2T reference genome was assembled did it become possible to resolve any genetic variation and detect molecular mechanisms involving repetitive regions [5]. The T2T human genome assembly covers centromeric, pericentromeric, and telomeric repetitive regions, sites which are frequently targeted by viral integrations, providing new opportunities to study as-yet unexplored repetitive regions and their functions in detail [83]. An increased detection rate for structural variations within centromeric and pericentromeric repetitive regions in cancer compared to previous assemblies was observed in the T2T assembly [5,6]. The T2T reference genome enhances the overall identification of structural variations not only in cancer genomes in general but also specifically help to resolve the complex structural variations of HPV integrants in repetitive regions of the genome. The T2T assembly extends the understanding of telomeres, centromeres, segmental duplications, and other complex regions, which are necessary to accurately map integration sites in the whole genome [5,6,68]. Since previous LR-Seq studies used the GRCh37 or GRCh38 reference genome (Table 1), it is likely that a significant proportion of integration sites in repetitive regions were missed. Additionally, previously identified integration sites often differ between studies and are supposed to be overestimated due to a lack of stringency in bioinformatic approaches for data analysis and missing validation by Sanger sequencing [84]. Molina et al. [38] noted that there is also a significant variability in the reported rates of HPV integration across different studies, which can be attributed to the differences in genetic backgrounds, methodologies, and sensitivities of the assessment technologies.

Nonetheless, LR-Seq is not a limitation-free technique. The costs are still relatively high, especially if high coverage is needed (HiFi), and the analysis requires high-performance computing centers and big data storage systems. The additional information on repetitive regions introduces more informational noise due to more variation that is evolutionarily more acceptable in non-coding regions.

## 8. Conclusions

The mechanisms guiding HPV integration remain debated, particularly regarding preferences for repetitive genomic regions, which are linked to genomic instability and carcinogenesis. However, a non-random integration mechanism is supported by the observation of recurrent integration hotspots across different HPV and tumor types. LR-Seq technologies (e.g., PacBio, Menlo Park, CA, USA and Oxford Nanopore, Oxford, UK) are especially suited for uncovering integration in repetitive regions, as they overcome limitations of SR-Seq, with comparable accuracy. LR-Seq can efficiently resolve complex integration structures, including large structural variations, interchromosomal translocations, and virus–human hybrid ecDNA while uncovering clonal evolution and intratumoral heterogeneity. While SR-Seq is still useful for broad population studies, LR-Seq holds greater clinical potential due to its ability to accurately capture full-length fusion transcripts and to detect epigenetic changes. The use of the T2T reference genome could further improve integration site mapping, particularly in previously inaccessible repetitive regions. High-resolution LR-Seq analysis will be critical for uncovering HPV-driven carcinogenic mechanisms and developing targeted therapies.

## Figures and Tables

**Table 1:** Comparison of selected studies on HPV integration using LR-Seq.

Cell Lines	Sequencing Method	Reference Genome	Integration Sites		Reference
			Hotspot Genes	Genomic Regions	
Cervical carcinoma	Oxford Nanopore, Oxford, UK	GRCh38	I: *MYC*, *KLF5*, *KLF12*, *TP63* M: *NR4A1*, *NR4A3*	Introns, repeats, exons, CpG islands	Porter et al., 2025 [26]
Cervical carcinoma, CaSki, HeLa	Oxford Nanopore	GRCh38	I: *RUNX2*, *CLIC5*	Introns, intergenic, exons, promotors, UTRs	Zhao et al., 2024 [61]
HeLa, SiHa	PacBio, Menlo Park, CA, USA Illumina WGS, San Diego, CA, USA	GRCh38	I: *KLF5*, *LINC00392*, *CCAT1*, *CASC21*	Intergenic, introns of ncRNAs	Wang et al., 2024 [49]
CaSki, HeLa, SiHa	Oxford Nanopore	GRCh37	None reported	None reported	Cui et al., 2023 [62]
OPSCC	Oxford Nanopore	GRCh38	None reported	Intergenic, introns, exons	Gauthier et al., 2023 [63]
Cervical carcinoma, HNSCC	Oxford Nanopore	GRCh38	M: *PIK3CA*, *STK11*, *CASP8*, *ZFHX3*, *RB1*, *HLA-A*, *FBXW7*, *KRAS*, *MYC*, *YAP1*, *RUNX2*, *BIRC2*, *BIRC3*	Intragenic, introns	Rodriguez et al., 2023 [64]
OPSCC, 93-VU-147T, GUMC-395, HeLa, HTEC	Oxford Nanopore PacBio HiFi	GRCh37	I: *EP300*, *MYC*	None reported	Akagi et al., 2023 [4]
Cervical carcinoma	PacBio Iso-Seq	GRCh38	M: *TP63*, *P3H2*, *GMDS-DT*, *CMAHP*	Intergenic, introns, exons, UTRs	Liu et al., 2023 [65]
Cervical carcinoma	Oxford Nanopore	GRCh37	I: *KLF5*, *LINC00392*, *CASC8*, *CASC21*, *MACROD2*, *TEX41*, *VMP1* M: *LINC00290*, *LINC02500*, *LENG9*, *IL20RB*, *SOX14*, *LENG8*, *LENG9*, *CDC42EP5*, *CASC21*, *CCAT2*, *CASC8*, *AKAP13*	Introns, intergenic, introns of ncRNAs, exons, UTRs	Zhou et al., 2022 [27]
Cervical carcinoma, CaSki	Oxford Nanopore	GRCh38	I: *CSMD3*, *ZFHX3*	Introns, intergenic	Yang et al., 2021 [66]
Cervical carcinoma	PacBio	GRCh37	M: *BNC1*, *RSBN1*, *USP36*, *TAOK3*, *NRAS*, *PVT1*, *TOP2A*, *SOCS3*, *GADD45A*	Introns, exons	Iden et al., 2021 [67]
Cervical carcinoma	Oxford Nanopore	GRCh37	I: *CHMP4B*, *RALY-AS1*	Intergenic, intros, introns of ncRNAs, exons	Yang et al., 2020 [68]

HNSCC: head and neck squamous cell carcinoma; OPSCC: oropharyngeal squamous cell carcinoma; ncRNA: non-coding RNA; UTR: untranslated region; I: integration; M: mutation (and deregulation); PacBio Iso-Seq: Pacific Biosciences isoform sequencing; HiFi: high fidelity.
